# The Monitoring of Psychosocial Factors During Hospitalization Before and After Cardiac Surgery Until Discharge From Cardiac Rehabilitation: A Research Protocol

**DOI:** 10.3389/fpsyg.2020.02202

**Published:** 2020-09-29

**Authors:** Edward Callus, Silvana Pagliuca, Enrico Giuseppe Bertoldo, Valentina Fiolo, Alun Conrad Jackson, Sara Boveri, Carlo De Vincentiis, Serenella Castelvecchio, Marianna Volpe, Lorenzo Menicanti

**Affiliations:** ^1^ Clinical Psychology Service, IRCCS Policlinico San Donato, San Donato Milanese, Italy; ^2^ Biomedical Sciences for Health, University of Milan, Milan, Italy; ^3^ Australian Centre for Heart Health, Melbourne, VIC, Australia; ^4^ Scientific Directorate, IRCCS Policlinico San Donato, San Donato Milanese, Italy; ^5^ Department of Cardiac Surgery, IRCCS Policlinico San Donato, San Donato Milanese, Italy; ^6^ Department of Cardiac Rehabilitation, IRCCS Policlinico San Donato, San Donato Milanese, Italy

**Keywords:** cardiac surgery, cardiac rehabilitation, psychosocial, psychocardiology, psychosocial risk factors, clinical psychology, heart disease

## Abstract

**Introduction**: There is considerable evidence that psychosocial factors contribute to the etiology and prognosis of cardiac illness. Currently, in Italy, psychologists are only obligatory in the cardiac rehabilitation setting, although there are indications that patients could be experiencing distress also during other moments of hospitalization, such as on admission for cardiac surgery.

**Objective and Methods**: The objective of this protocol is to gain more information about cardiac patients, specifically during the various moments of hospitalization for cardiac surgery, by collecting data at admission before cardiac surgery (t0), at admission to cardiac rehabilitation (t1), and at discharge (t2) at the Istituto di Ricovero e Cura a Carattere Scientifico (IRCCS) Policlinico San Donato hospital. A psychosocial questionnaire was constructed after consulting the relevant national and international guidelines. Patients admitted for cardiac surgery and attending a rehabilitation program will be evaluated by acquiring data about their civil status, religiosity, education and work capacity, social condition (including the presence and quality of intimate relationships and support received), previous psychological and psychiatric histories, psychological status, lifestyle (including questions on nutrition, smoking, alcohol, and substance abuse), adherence to therapy, quality of life (QoL), health perception, anxiety, and depression at t0. Health perception, anxiety, and depression are also measured at t1 and t2.

**Discussion and Conclusion**: This study is an attempt to identify the recommended psychosocial variables which need to be monitored during cardiac patients’ hospitalization for cardiac surgery, through to the completion of cardiac rehabilitation. After implementing this study at the IRCCS Policlinico San Donato, attempts will be made to create studies on a national and international level to generate more evidence regarding these variables, in order to create tailor-made interventions for these patients during these specific and delicate moments.

## Introduction

The leading cause of deaths in the latest National Vital Statistics Report to date in 2017 was “Diseases of the heart” (Murphy et al., 2018). Anxiety, depression, and stress are among the most important psychological risk factors related to heart disease and are predictive of mortality and/or of a worsening of the quality of life (QoL) (Hare et al., 2013).

The most recent 2016 European Guidelines for Cardiovascular Diseases reported that a series of psychosocial factors, including low socio-economic status, lack of social support, stress at work and in family life, hostility, depression, anxiety, and other mental disorders are associated with the risk of developing cardiovascular disease (CVD). In addition, when these factors are absent there is a lower risk of developing CVD and a better prognosis of CVD. Psychosocial risk factors are also linked to treatment adherence, type of lifestyle, and the promotion of health in patients and populations. For this reason, it is recommended that assessment of psychosocial risk factors with the use of clinical interview or standardized questionnaires is carried out (Piepoli et al., 2016).

The biopsychosocial model (Engel, 1977) was further elaborated in the field of cardiac illness with the development of psychocardiology, which can be defined as an effort to see how psychology can contribute in the prevention, treatment, and rehabilitation of patients with cardiac disease (Molinari et al., 2006). The relevance of the multidisciplinary team (Tramarin et al., 2007; Griffo et al., 2008) and also economic benefits of psychological intervention in hospitals have been highlighted, with effective teams providing increased patient safety and better information sharing about the patients (Bettinardi et al., 2014; Gilardi et al., 2014).

In the paper “Best practice in psychological activities in cardiovascular prevention and rehabilitation: Position Paper” (Sommaruga et al., 2018), psychologists of the Italian Association for Cardiovascular Prevention, Rehabilitation and Epidemiology (GICR-IACPR) reviewed the key components of psychological activities in cardiovascular prevention and rehabilitation (CPR). The existence of an association between depression, anxiety, social factors, stress, personality and illness onset/outcome, and coronary heart disease (CHD) was confirmed. When it comes to interventions, cognitive-behavior therapy, interpersonal therapy, and short-term psycho-dynamic therapy are the main ones proposed, together with psychoeducational initiatives. An interesting factor, which is highlighted, is the analysis of the family/caregivers and their need for psychological support. Today, many examples in the literature show the positive effects of good family and social support on the outcome of CVD in terms of better adherence and a decrease in new hospital admissions (Sommaruga et al., 2018).

Following a review of the national and international guidelines and operational instructions in force from 2018 in various national and international jurisdictions, the “Minimal Care” requirements for clinical practice have been identified for individual professionals constituting a cardiological rehabilitation team (Fattirolli et al., 2018). In these requirements, it was specified that when it comes to psychological care, during the assessment phase, both a clinical interview and standardized tests can be utilized covering the areas of psychological functioning, perceived social and family support, disease awareness, and motivation for treatment. When it comes to possible interventions, the clinical interview, relaxation techniques, inclusion of the caregiver, involvement of community welfare services in the case of social issues, psychoeducation, and collaboration with the multidisciplinary team are suggested.

In the German heart society clinical commission (DGK) position paper, it is recommended that in clinical practice for patients with acute and chronic CHDs, there is a need for psychotherapeutic and psychological support for these patients since affective disorders [e.g., depression, anxiety, and post-traumatic stress disorder (PTSD)] are often noticed in patients with cardiac problems (Ladwig et al., 2014).

In a 2018 update, based on the results of a systematic review of evidence-based guidelines and clinical experiences, it was found that psychosocial variables such as low socio-economic status, acute and chronic stress, depression, anxiety, and poor social support are associated with an unfavorable prognosis. According to these data, it is advisable to systematically evaluate both the psychosocial aspects and the cognitive function of all patients as a part of routine clinical assessment. In all cases where critical values emerge from the evaluation, continuing psychosocial and psychotherapeutic interventions and/or psychopharmacological interventions should be carried out since the clinical evidence has shown that such interventions are positive for the prognosis of patients suffering from CHD, chronic heart failure, arterial hypertension, and some arrhythmias (Albus et al., 2019).

There has been a lot of attention paid to psychosocial aspects in cardiac rehabilitation guidelines, and on hospitalized patients (Piepoli et al., 2016; Fattirolli et al., 2018; Sommaruga et al., 2018). In the next paragraphs, we report studies in which psychosocial factors were investigated in the pre-operative phase, and other studies in which longitudinal research was carried out, in order to further consolidate the basis of this psychosocial research protocol and questionnaire.

## Cardiac Patients Psychological and Psychosocial Variables

When it comes to cardiac diseases, we need to take into consideration the behaviors that are linked to health outcomes, such as physical inactivity, smoking, poor dietary habits, and stressful behaviors, which are difficult to change. Most social-cognitive theories assume that an individual’s intention to change is the best direct predictor of actual change. A discrepancy between intention and behavior is normally seen in these patients, however. The health action process approach (HAPA) suggests a distinction between the pre-intentional motivation processes that lead to a behavioral intention and post-intentional volition processes that lead to the actual health behavior (Schwarzer, 2008). For this reason, the psychosocial questionnaire will be utilized in order to make an initial assessment and increase the awareness of the patients on these variables in order to facilitate the adoption and maintenance of health behaviors in the post-hospitalization phase.

The pre-surgery phase is considered a very delicate one since patients are at particular risk for psychological vulnerability. Patients who undergo cardiac surgery, in fact, are prone to emotional imbalance, distress, and fear that in many cases may result in anxiety and depression (Gomes et al., 2019).

This phase also represents a possibility of cure, but at the same time also of failure. The fear of the unknown, coupled with the likelihood of failure, could worsen the patient’s state of anguish. Often this is associated with the risks, uncertainties, limitations, and pain that come with it. Initially, the intervention is seen and perceived by patients as something “magical” that could free them from pain and suffering, while during the hospitalization, they experience states of anxiety, depression, stress, and other negative feelings (Gomes et al., 2019).

### Anxiety and Depression

When it comes to anxiety and depression, surgery can represent a real trauma and is often associated with a high level of anxiety. Pre-operative anxiety is defined as a state of discomfort or malaise secondary to the pathology and the hospitalization. A high pre-operative level of anxiety increases the risks associated with surgery, including morbidity, mortality, and the recovery process (Frazier et al., 2003).

In recent decades, there has been a high level of recognition of the significant relationship between depression and CVD with 20–25% (Andrew et al., 2000; Tully and Baker, 2012; Murphy et al., 2016) of patients undergoing heart surgery reporting high rates of severe depression. While depression resolves for many patients, pre-operative depression states have been shown to persist even after surgery, increasing the risk of cardiac morbidity or mortality (van Melle et al., 2004; Murphy et al., 2013; Patron et al., 2014). One possible reason for the association between depression and cardiac morbidity is that patients with depression are less likely to adhere to medical care and participate in cardiological rehabilitation programs (Borowicz et al., 2002). In addition, physiological mechanisms involved in the associations between mental illness and CHD have been well-documented in several reviews published in recent years (Jackson et al., 2018). Some examples of these are: (a) hypothalamic pituitary adrenal axis dysregulation, (b) platelet activation, and (c) inflammation (Brydon et al., 2006; Smith and Blumenthal, 2011; Goldstein et al., 2015). Patients with depression have higher levels of biomarkers that promote atherosclerosis; in anxiety and depression, we see reduced heart rate variability, suggesting decreased parasympathetic activity, altered serotonergic pathways, altered platelet aggregability, and increased C-reactive protein, an indicator of increased inflammatory response.

A study by Gardner (Gardner and Worwood, 1997) detected high levels of anxiety and depression in patients undergoing heart surgery, both in the pre-operative and post-operative phases. The study showed that a psychological assessment and intervention can be useful and contribute to a better prognosis. It has been observed that the same pre-operative mood could persist after surgery, and for this reason, the mood tone is often used as a predictive indicator of anxiety-depressive symptoms in the post-operative phase (Magni et al., 1987).

In a study by Gallagher and McKinley (2007), 172 patients admitted for coronary artery bypass surgery were interviewed in order to determine their major concerns and anxiety level at three different times: before surgery, before discharge, and 10 days after discharge. The hospital anxiety and depression scale (HADS) and the stressor scale were used. The results showed that the Cardiac Surgery Unit represents a very stressful environment for patients waiting to undergo cardiac surgery, as well as for their families. Major concerns reported by the patients were related to the surgery and its possible consequences, such as fear of death, being away from home or work and experiencing pain. It is noticeable that being away from home caused feelings similar to those related to physical pain.

In this study, many patients had traveled extensively to reach the hospital to undergo this surgery. The distance variable had consequences not only for the patient who perceived it as a moment of discontinuity, but also for the families who had to quickly adapt to this new condition. Concerns about the economic and the general family management spheres were identified as some of the causes of depressive and anxious symptoms in these patients (Gallagher and McKinley, 2007), as has been reported more recently (Murphy et al., 2019). A high level of pre-operative anxiety has been shown to be correlated with the presence of persistent post-operative pain (Choinière et al., 2014) while a study by Andrew et al. (2000) found that the degree of pre-operative and post-operative depression and anxiety is related to post-operative neuropsychological dysfunction. High levels of anxiety and depression at both pre-operative and post-operative assessments are predictive of attention and verbal memory impairment, which are the cognitive domains known to be affected by mood tone.

Another study showed that patients with a history of depression are exposed to a higher risk of developing post-operative infections (Theodore et al., 2019). These results confirm findings of Scheier et al. (1999) evaluating re-hospitalization rates due to sternal wound infections in association with depression and risk of re-intervention. In conclusion, we can say that depression and anxiety are predictive and detectable factors with standard and objective assessments, which can negatively affect post-operative recovery. The identification and the treatment of these factors in the pre-operative phase could represent an enhancement to improve care, decrease patient hospitalization, and improve overall patient well-being.

### Quality of Life and Health Perception

Heart surgery is an important stressful event that can have a negative effect on patients’ QoL and can cause post-traumatic reactions from acute stress and cumulative long-term stress. The concept of health-related quality of life (HRQoL) is a multidimensional concept linked to the self-perception of physical, emotional, mental, and social well-being. It indicates both the functional impact of the disease and at the same time, the overall satisfaction of the patient undergoing the intervention (Gražulytė et al., 2019).

The correlation between HRQoL, marital status, and various comorbidities has been assessed in a sample of men and women undergoing heart surgery. Results showed that women have a slower recovery and lower HRQoL results compared to men after heart surgery. This suggests the importance of considering both gender and the presence of social support as factors that can affect the recovery of patients from heart surgery. The study showed the need for follow-up and support especially for women who live alone (Bjørnnes et al., 2017).

### Social Support, Loneliness and Intimate Relationships

It has been noted that being married or having a partner is associated with a lower risk of developing heart disease. Living alone, on the other hand, could be an indicator of a particularly stressful psychosocial situation in terms of isolation or lack of social support (Murphy et al., 2008; Hagström et al., 2018).

Therefore, it would be appropriate to investigate the social situation and the quality of social support of patients undergoing heart surgery. It has been noted that poor and ineffective social support increases the risk of post-operative depression in patients over the age of 55 (Oxman et al., 1994; Jackson and Murphy, 2019).

Loneliness has been associated with significantly poorer patient-reported outcomes across various cardiac diagnoses, and in addition has been shown to predict all-cause mortality 1 year after the cardiac event in both men and women (Christensen et al., 2020). The importance of the perception of the quality of support in this population is also linked to patient-reported outcomes on mood and health behaviors (Vilchinsky et al., 2011).

### Pharmacological Adherence

Poor adherence to medications can cause recurrent problems, high morbidity, and higher mortality rates. Many factors contribute to poor adherence, including low self-efficacy and depression. The use of technology may be considered as an effective tool to promote good adherence, as it promotes self-monitoring strategies, education, and greater knowledge (Park et al., 2015).

### Smoking

Smoking is linked to high mortality along with alcohol abuse, low physical activity, and poor diet. Cigarette smoking contributes to the development of coronary artery disease and myocardial infarction. Furthermore, persistent smoking after cardiac surgery is considered a predictor of the possibility of increased mortality. Studies on elderly populations admitted to cardiac surgery have indicated smoking as a significant risk factor (Jones et al., 2011).

A meta-analysis showed that smoking leads to an increased level of mortality while a decrease in smoking in the post-operative phase improves duration of life expectancy. The period prior to cardiac surgery is a favorable period for educating patients about smoking damage, especially because the relationship between smoking and CVD is not evident for many patients (Bayfield et al., 2018).

### Substance Abuse

The use of drugs is associated with significant cardiovascular complications, including cardiomyopathies (Awtry and Philippides, 2010). The current guidelines of the American college of cardiology for the management of acute coronary syndromes (ACSs) recommend a toxicological screening of urine when substance abuse is suspected. It is known that cannabinoid receptors are present in myocardial muscle cells, and it has been shown that the use of marijuana is associated with adverse cardiovascular outcomes, including stroke, coronary artery dissection, ACSs, and cardiomyopathies (DeFilippis et al., 2018). Ischemia and acute myocardial infarction are the most frequently described diseases seen in cocaine abuse while several studies describe the link between abuse of methamphetamine and cardiomyopathies (Segawa et al., 2019; Tiako et al., 2019; Wang et al., 2019). It has been found that the use of cocaine causes a series of pro-thrombotic alterations and as well as myocardial infarction with intravenous users more likely to develop episodes of endocarditis and myocarditis (Badarienė et al., 2019).

### Alcohol Consumption

Moderate alcohol consumption is associated with a lower risk of CHD, and an association between moderate and low morbidity and mortality among patients with recent myocardial infarction has also been found (Maclure, 1993). A study of 1,919 patients undergoing aorto-coronary bypass assessed the association between alcohol consumption and mortality, showing that heavy drinkers showed an increased risk of mortality (Grabas et al., 2016).

### Level of Education

In a study of modifiable risk factors, CVD, and mortality in a large sample, behavioral risk factors contributed most to deaths although the single largest risk factor was a low education level. The level of education is strongly associated with a high risk of mortality due to the its association with health literacy and patient activation (Yusuf et al., 2019).

Recently, some studies have shown that post-operative delirium is associated with increased cognitive impairment and a higher incidence of dementia up to 15 months after surgery (Sprung et al., 2017).

### Quality of Sleep

Sleep represents another predictive factor of rapid recovery from surgery. A study published in 2013 highlighted that the presence of sleep disorders prior to aorto-coronary bypass surgery may partly explain why some patients fail to have adequate and comprehensive physical recovery (Poole et al., 2013).

There is a high prevalence of obstructive sleep apnea (OSA) in ACS patients that persists within the first 6 weeks following hospital admission (Garcia-Rio et al., 2013; Nakashima et al., 2015). Studies have shown that close to two-thirds of ACS patients exhibit mild to moderate OSA. Even mild levels of OSA with minimal daytime symptoms are associated with adverse effects on endothelial function and hypertension (Kohler et al., 2008; Li et al., 2017). In the Nakashima (2015) study, close to half of all patients were diagnosed with moderate to severe OSA, a level that has been independently associated with 2.3 times the risk of recurrent ACS events after adjusting for covariates, such as age, sex, body mass index (BMI), smoking, and diabetes.

### Religiosity

Religiousness refers to subjective attributes, such as religious doctrine and behaviors, including praying and participating in religious initiatives. In a prospective study, the influence of religiosity, as another psychosocial factor, was assessed to measure the impact on patients undergoing surgery (Contrada et al., 2004).

Fear of death associated with heart surgery can increase spiritual beliefs. Such beliefs can offer patients feelings of comfort and provide them with a means to address these fears and concerns related to surgery. Ai et al. (2002) found evidence that praying during the period before surgery was associated with a high level of optimism the day before the procedure.

Stronger religious beliefs are associated with lower surgical complications and shorter hospitalization (Contrada et al., 2004).

### Working and Socio-Economic Status

Changes in the working environment can be a cause of stress causing psychological repercussions and potentially having an impact on the post-operative recovery of patients. It would therefore be appropriate to investigate in the pre-operative phases: the type of work of the patient, the level of work stress, the required physical and mental efforts, and job satisfaction. These are considered to be important elements (Hanson et al., 2019) as low socio-economic status has an effect comparable to that of the main risk factors for the development of CVD and post-operative recovery (Stringhini et al., 2017).

### Nutrition

The role of nutritional behaviors and dietary intake in the prevention of CVDs or in its progression has been extensively studied. Numerous studies have highlighted the importance of healthy dietary choices and improved lifestyle with a higher level of physical activity and increased intake of healthy food choices, including fruits and vegetables and food antioxidants for the prevention and treatment of cardiovascular events (Farhangi and Najafi, 2018).

Older patients undergoing cardiac surgery show greater prevalence of comorbidity with malnutrition being a determining factor for the dysfunction of many organs.

Adequate nutritional therapy has been suggested to improve patient outcomes through maintenance of energy metabolism, intestinal integrity, microbial diversity, and better wound healing. Pre-operative nutritional status and post-operative nutritional management can be important factors for clinical outcomes in patients undergoing cardiac surgery who are at high nutritional risk.

Malnutrition has been reported to increase morbidity and mortality after cardiac interventions. Several researchers have reported a correlation between a poor diet and an increase in complications and negative results following a heart surgery. Many studies (Engelman et al., 1999; van Venrooij et al., 2008, 2012; van Straten et al., 2010) suggest that nutritional status should be a factor to be evaluated, even in the pre-operative phase, to determine whether patients are selectable for surgery. Although obesity may be associated with a high risk in cardiac surgery, a study by Loop et al. (1983) suggested that paradoxically, lean patients might actually have a higher risk after coronary operations than obese patients (Sanchez et al., 2011).

### Physical Activity

Many studies have shown that adequate physical activity and exercise-based cardiac rehabilitation have a positive effect on the recovery of CHD patients. This activity improves cardio-respiratory function and reduces cardiac mortality as well as shortening length of hospital stay (Warburton et al., 2006).

In a 2017 study, it was noted that a reduction in physical activity represents a negative predictive factor for post-cardiac intervention outcomes for patients over the age of 65 (van Laar et al., 2017). In an Australian study, physiotherapists monitored the physical activity of the patients from pre-hospitalization up to the day before heart surgery. It was shown that the activity monitored and followed by a physiotherapist promotes the improvement of post-operative functional and physiological capacities, reducing the length of hospitalization (Mungovan et al., 2017).

It can be seen that the assessment and treatment of psychosocial risk factors improve the QoL and provide an ethical obligation to provide comprehensive and effective psychosocial care (Gallagher et al., 2015).

## IRCCS Policlinico San Donato: an Italian Example

In 2007, in Italy, the Italian SurveY on carDiac rEhabilitation-2008 (ISYDE-2008; Tramarin et al., 2007) showed an imbalance between the request and the offer of cardiac rehabilitative intervention, encouraging the development of organizational models suitable for ensuring for all patients in the stage of chronicity, a unique process of preventive and rehabilitative cardiology. This led to the drafting of a second survey that provided a complete overview of the available activities in cardiac rehabilitation (Griffo et al., 2016). In this multicenter survey, it was reported that there was an incremental trend of cardiac rehabilitation provision in Italy.

Since 2013, at the IRCCS Policlinico San Donato, the units of cardiac surgery, cardiac rehabilitation, and the Clinical Psychology Service were structured based also on these services. It is unusual to have both cardiac surgical and cardiac rehabilitation of a considerable size in the same hospital structure, and this provides a good opportunity to identify the psychosocial characteristics of acquired cardiac patients from the pre-surgery phase up to the stage of discharge from cardiac rehabilitation in the case of patients who continue their rehabilitation process in the same institute.

Another interesting consideration is that in Italy, the psychologist is legally obligatory only in the cardiac rehabilitation setting and not in the cardiac surgery and other settings. In order to effectively implement the guidelines, 0.5 psychologists are suggested for every 12 patients in institutes, which treat 275 patients a year (Griffo et al., 2008).

Along with the national recommendations, there are also regional recommendations. When it comes to psychological activity in cardiac rehabilitation, the inclusion of a psychologist in the multidisciplinary team is calculated on the basis of the total number of minutes which each patient must complete with the multidisciplinary team. It is not specified that the patient must do a certain number of minutes per week only with the psychologist, but that each patient must do a total of at least 500 min per week with the following professional figures: physiotherapist, occupational therapist, speech therapist, neuro-psychomotor therapist, educator, psychologist, and dietician (Jayasinghe et al., 2009).

From a clinical point of view, the psychosocial evaluation requested in the cardiac rehabilitation multidisciplinary setting should now be provided in the cardiac surgery setting. The patients would be seen for an initial evaluation and at least for another two sessions (t0, t1, and t2). During the first evaluation, it is established if more sessions will be required.

In order to construct and update the psychosocial questionnaire administered during admission, the pertinent national and international medical and psychosocial guidelines were consulted (Griffo et al., 2008; Piepoli et al., 2016; Fattirolli et al., 2018; Pedretti et al., 2018; Sommaruga et al., 2018). In addition, the pertinent literature addressing the areas indicated by the guidelines were further explored.

## Study Research Protocol

The Clinical Psychology Service of the IRCCS Policlinico San Donato created in August 2016 has developed the following study protocol, which has been approved by the Ethics Committee of the San Raffaele Hospital in Milan on the 16th of September 2020 (registration number 159/int/2020), which will be conducted according to the principles expressed in the Declaration of Helsinki, the institutional regulation and Italian laws and guidelines. Written informed consent will be obtained from the patients.

This study is partially supported by Università degli Studi di Milano through the Open Article Process Charges (APC) Project and the Ricerca Corrente funding from the Italian Ministry of Health to IRCCS Policlinico San Donato. The authors have no competing interests to declare.

The study is a longitudinal observational prospective study aimed at detecting the psychosocial variables of patients admitted to the IRCCS Policlinico San Donato, which includes the units of cardiac surgery, cardiac rehabilitation, and the Service of Clinical Psychology. This organizational set-up is almost unique in Italy, allowing psychologists of the Clinical Psychology Service to contact all cardiac patients before heart surgery and follow them until their discharge from hospital.

This unique organizational condition enables the identification of the psychosocial characteristics of acquired heart patients, from the moment they are admitted for heart surgery to their discharge from cardiac rehabilitation in our institute.

### Objective of the Study

#### Primary Outcome

The main objective of the study is to measure health perception (EuroQoL VAS 0-100), anxiety (GAD-7), and depression (PHQ-9) before cardiac surgery (t0) and at discharge from cardiac rehabilitation (t2) and to identify the medical and psychosocial variables which influence these scores.

#### Secondary Outcomes

Health perception, anxiety, and depression will be assessed also at discharge from cardiac surgery or admittance to cardiac rehabilitation (t1) to explore possible trends.

In addition, the evaluation of possible correlations between all other psychosocial factors, cardiac diagnosis, ejection fraction (EF), duration of hospitalization, and the performance of the 6 min walking test (6MWT), which is done twice by the physiotherapists during the admission in cardiac rehabilitation and just before discharge (t1 and t2) and the New York heart ability index (NYHA) administered during all times (t0, t1, and t2) will be carried out.

#### Study Design

This is a longitudinal observational prospective study.

### Patient Selection

#### Target Population

Patients with acquired heart disease admitted to heart surgery, who will undergo heart surgery, either through a sternotomy or with minimally invasive surgery, who will also be admitted to the cardiac rehabilitation operative unit in the same institution, after surgery will be included in this study. Most patients hospitalized in our center undergo elective surgery for heart valve repair or replacement followed by those who undergo coronary artery bypass surgery. Heart surgery patients undertake a 20-day cardiac rehabilitation program (CRP) either at IRCCS Policlinico San Donato or in another CRP, sometimes closer to the residence of the patients. Only in the case of minithoracotomy or transcatheter aortic valve implantation (TAVI), or transcatheter aortic valve replacement (TAVR) in most of the cases, no CRP is needed.

The number of psychological sessions given and their classification according to the taxonomy of behavior change techniques (Abraham and Michie, 2008) will be taken into consideration in the analysis. For example, for patients who report high anxiety and depression, the following interventions could be indicated: prompt self-monitoring of behavior, agreement on behavioral contract, and stress and time management. In the case of a lack of adherence and the presence of behaviors linked to risk factors, such as smoking, motivational interviewing, and prompt behavioral goals could be recommended. Finally, in the cases of social isolation, provision for opportunities for social comparison and the planning of social support or social change could be enacted by facilitating the referral of the patients to an adequate center, where they are resident once they are discharged.

#### Inclusion Criteria:

1. The patients’ age is ≥18 years.
2. The patient is being treated at the Cardiac Surgery Unit of the IRCCS Policlinico San Donato.
3. The patient will undergo cardiac surgery.
4. The patient is able to understand the condition of the study.

#### Exclusion Criteria:

Patients with:

1. Cognitive impairment.
2. Diagnosis of a severe psychopathology with impaired reality data.
3. Genetic syndrome.
4. Language barriers.

### Study Procedure

The questionnaire is given to the patient by the staff of the Clinical Psychology Service, and they are instructed to complete it on their own. It is specified to the patients that if they encounter any difficulties the clinical psychology staff will help them to complete it, and that there will be an initial session in which it will be discussed, in order to decide what kind of support is required.

The Clinical Psychology Service team will be informed about admitted and discharged patients and their medical conditions by participating in the daily medical morning briefings and by consulting the internal patient management system (Galileo e-Health Solutions).

All patients being admitted to the Cardiac Surgery Unit will be eligible for inclusion in the study if they meet the inclusion criteria.

Times of Administration of the Psychosocial Questionnaire:

1. At time zero (T0), pre-operative phase, the administration of the full questionnaire and validated tests.
2. At time one (T1), after a minimum 7 days from T0 (post-operative phase or admission to cardiological rehabilitation), administration of the following tests: PHQ-9, GAD-7, and EuroQoL-VAS.
3. At time two (T2), cardiac rehabilitation discharge administration only of the following tests: PHQ-9, GAD-7, and EuroQoL-VAS.

At the time of admission to the Cardiac Surgery Unit, it is not possible to foresee if the patient will perform their rehabilitation program at IRCCS Policlinico San Donato; however, all patients will still be enrolled in the study so that the psychosocial assessment will be useful to support the patients throughout the course of hospitalization especially if critical areas should emerge. In addition, the data that will be collected at T0 and T1 will be useful to define a psychosocial profile of patients undergoing cardiac surgery.

### Study Variables

The following medical and functional variables will be considered: cardiac diagnosis, ejection fraction (EF), duration of hospitalization, and the performance of the 6MWT.

The variables taken into consideration from the guidelines and the other pertinent literature can be summarized in the following Table 1. Each variable will be described in detail in the following paragraphs.

**Table 1:** Variables and measurement tools.

Variables	Measurement tools	# items
Age and gender	Determination of age and gender from medical records	2
Citizenship	Indication if citizenship is Italian or Foreign (with specification of citizenship)	1
Religion	Indication of religion and level of religiosity	2
Civil status	Indication of civil status (single, married, widowed, legally separated, or divorced)	1
Education level	Indication of the level of education obtained and the total number of years of studying	2
Occupation and retirement	Items on type of occupation [part time or full time, unemployed, looking for a job, housewife, disability and percentage of disability, student, retired, or other condition (with specification)] Indication on type of work (mental or physical). satisfaction with work, level of stress perceived, and capacity to work In case of retirement, satisfaction regarding the previous working years and enquiry whether the patients have found a satisfactory way of employing their time	8
Social condition, presence and quality of a close relationship, and perceived support	Marital status or presence of intimate relationship Evaluation of the quality of the relationship Number of children Enquiry about cohabitation status and with whom the patients live Enquiry about perception of receiving adequate support specifically during the moment of enquiry	5
Psychological and/or psychiatric history	Previous psychological and psychiatric visits and reasons for them. Past psychological and psychotherapeutic treatment and utilization of psychotropic drugs	5
Nutrition	Items regarding whether appetite is normal and about eating habits	2
Quality of sleep	One item regarding sleep quantity and quality	1
Physical activity	One item regarding the amount of physical exercise outside of work	1
Smoking addiction	Fagerström test	8
Alcohol consumption	One item exploring alcohol consumption	1
Substance abuse	Items on current and past drug use and in case of a positive answer, an enquiry about the intention of stopping	3
Gambling	One item on the presence and frequency of gambling	1
Therapy adherence	Morisky, Green, and Levine (MGL) adherence scale	4
Life satisfaction scale	Satisfaction with life scale (SWLS)	5
Psychological functioning (Anxiety and depression)	Patient health questionnaire-9 (PHQ-9) General anxiety disorder-7 (GAD-7)	16
Perceived health	Visual analog scale (EuroQoL-VAS)	1
Quality of life	Visual analog scale quality of life (QoL-VAS)	1
Health questionnaire	The short form (12) health survey	12
∑ items		82

### Assessment Tools

General patient history-in the initial part of the questionnaire, the following demographic and social variables are explored: marital status, presence/absence of children, culture of origin, education level, occupation situation, and religion.

Patients are also asked if they are recieving social support and if they feel it is adequate, if they feel lonely, if they have a partner and to specify the quality of this relationship. Although numerous scales have been developed to assess social support in this population, no standard scale has gained widespread acceptance in clinical practice, and it was indicated that enquiring about who the patients turn for social support is sufficient in clinical practice (Rozanski, 2016).

Furthermore, they are asked information about physical activity, alcohol and drug use, and other addictions. Active smokers are asked to complete the Fagerström questionnaire in order to identify the degree of nicotine dependence. *The Fagerström test* (Heatherton et al., 1991) is a self-administered test that evaluates the current dependence on nicotine. The original 1978 questionnaire was simplified and further developed. It consists of six items, the different answers of which allow a classification of nicotine addiction. Total score ranges from 0 to 10, 10 being the highest level of addiction and 0 indicating the lowest level of nicotine dependency. The test was copyrighted in 1991, but can be used without permission. This instrument has also been utilized to measure nicotine addiction in the CHD population (Papazisis et al., 2019).

#### Morisky, Green, and Levine Adherence Scale

This is the most widely used questionnaire on adherence to drug therapy (Morisky et al., 1986). It is a one-dimensional questionnaire that contains four items, and the 1986 version does not require a license for usage. The total score ranges from 0 to 4, and a score lower than 3 indicates a bad adherence to drug therapy. This version of the test was utilized to assess the adherence to cardiovascular medicines (Santra, 2015).

#### Satisfaction With Life Scale

This is a short tool, which takes only 1 min to complete; it consists of five items that measure the overall cognitive assessment of life satisfaction experienced by the patient (Broadbent et al., 2006). Patients are asked about their level of agreement/disagreement with each of the five options, indicating for each statement the corresponding number on a seven-point Likert scale ranging from 7, very much in agreement to 1, strongly disagree. The total score ranges from 0 to 35, 0 indicating the least satisfaction with life and 35 indicating the highest satisfaction with life. The scale shows good convergent validity with other scales that evaluate subjective well-being. Life satisfaction, as assessed by the satisfaction with life scale (SWLS) test, shows some stability over time and has shown sufficient sensitivity in the detection of change and life satisfaction during a clinical intervention. Since the scale evaluates the patient’s conscious and subjective judgment on his life, it is advisable to use this tool in a complementary way to other tests focused on psychopathology and emotional well-being to capture more unconscious and latent aspects. This tool has been copyrighted since 1985 but the authors, Ed Diener, Robert A. Emmons, Randy J. Larsen, and Sharon Griffin, have allowed its use without a request for authorization and without charge (Diener et al., 1985). This test has been previously utilized in the cardiac population (Gao et al., 2019).

#### Patient Health Questionnaire-9

This is a short psychological screening tool designed to measure symptoms of depression in primary care facilities (Kroenke et al., 2001). The patient health questionnaire-9 (PHQ-9) test is completely free for healthcare professionals. Pfizer Inc., the legal copyright holder, explicitly states that “no authorization is required to reproduce, translate, view, or distribute the PHQ-9.” It contains nine questions that help identify patients with clinically significant symptoms of depression. Patient responses are rated 0–3 with 0 representing “not at all” and 3 indicating “almost every day;” the PHQ-9 contains a total score range of 0–27, with a higher score meaning a higher level of depression. In addition, it has been utilized for the screening of depression in the cardiac population (Berge et al., 2019).

#### Generalized Anxiety Disorder

This is a valuable screening tool for generalized anxiety disorder in clinical practice and research (Kroenke et al., 2007). In addition, it provides a measure of severity and is linked to the criteria of the diagnostic and statistical manual of mental disorders (DSM-IV). The general anxiety disorder-7 (GAD-7) can be particularly useful in assessing the severity of symptoms and monitoring changes over time, although its responsiveness to changes remains to be tested in treatment studies (Spitzer et al., 2006). GAD-7 has been evaluated as a reliable tool for detecting the most common anxiety disorders. There was a specificity of 82% for GAD. It is moderately effective in panic disorder (PD) screening (sensitivity-74% and specificity-81%), social anxiety disorder (sensitivity-72% and specificity-80%), and PTSD (sensitivity-66% and specificity-81%). Total score ranges from 0 to 21, with a higher score indicating a higher level of anxiety and the presence of severe anxiety when scores are equal to or higher than 15. Permission for use, replay, and deployment is not required. The GAD-7 has been utilized for the screening of anxiety in cardiac patients (Berge et al., 2019).

#### EuroQoL-VAS

This is the second part of the EuroQoL-5D questionnaire, a standardized tool that measures the health of respondents and their QoL on the basis of which it is possible to assess their health care, a technique, and a technology (EuroQol Group, 1990). It consists of a visual analog scale (VAS) of 20 cm, graphically represented as a graduated thermometer, on which the patient indicates the best (score-0) or worst (score-100) possible state of health. In addition, its psychometric properties were considered adequate for hospitalized and rehabilitation cardiac patients (Schweikert et al., 2006; Quanjel et al., 2019).

#### QoL-VAS

The term QoL generally refers to the well-being (emotional and social) of an individual and the patient’s ability to fulfill the tasks of daily life satisfactorily (Moons et al., 2006). It consists of a VAS of 20 cm, graphically represented as a graduated thermometer, on which the patient indicates the best (score-100) or the worst (score-0) about the perception of own QoL. A VAS is the recommended method to evaluate overall QoL. This type of scale has been utilized to assess QoL in patients with congenital heart disease (Apers et al., 2015).

#### The Short Form (12) Health Survey

The short form-12 (SF-12) questionnaire is the abbreviated version of the “Short Form 36 items Health Survey” (SF36) and serves as a generic indicator of QoS and assesses the individual’s subjective perception of the concept of health (Apolone et al., 1997). Through the 12 questions proposed in the test, SF-12 investigates eight aspects of health: physical activity, limitations due to physical health, emotional state, physical pain, perception of general health status, vitality, social activities, and mental health. The synthesis of total scores allows you to build two synthetic indices, a physical health index-12 (PCS-12) and a mental health index-12 (MCS-12). The lower the score of the two indices (indicatively below 20 points), the higher the level of disability. The SF-12 is a valid questionnaire (Apolone et al., 1997), which is reliable and suitable for self-administration, even in the case of geriatric users and has been used in cardiac populations (Quanjel et al., 2019) and in a 2009 study of a sample of heart patients before and after 3 months of surgery (Juergens et al., 2010).

### Statistics

Categorical variables will be shown as count and percentage, normal variables as mean ± SD, non-normal variables as median and interquartile range. Normality assumption will be tested in continuous variable by visual inspection of qq-plot. Correlations between continuous variables will be evaluated according to Pearson’s R or Spearman’s Rho, depending on the distribution. *t*-test or Wilcoxon rank sum test will be used to compare unpaired means in normally or non-normally distributed variable, respectively. Fisher’s exact test will be used to compare categorical data between groups. Change at discharge from cardiac rehabilitation (t2) from before cardiac surgery (t0) will be analyzed to assess the treatment effect on measure health perception (EuroQoL VAS 0-100), anxiety (GAD-7), depression (PHQ-9), and the identification of medical and psychosocial variables, which influence these scores using ANCOVA analysis.

Based on previous clinical experience, the minimal expected intra-subject difference before/after rehabilitation should be in depression with a score = 1.5 with a standard deviation of 5.0. Therefore, a sample size of 155 patients would be required, using a two-sided paired Wilcoxon signed-rank test, *α* = 0.01 and a power of 0.90. We decide to extend the sample size to 310 patients considering the possibility of a 50% dropout, which would be comprised of the patients who will be hospitalized in other institutions for cardiac rehabilitation, most often due to logistical reasons (they would be closer to home in these institutes).

## Discussion

In recent years, psychology has strengthened the link between psychological factors and organic pathologies, giving rise to new disciplines, such as psychocardiology or cardiac psychology as it is also known, which brings to light the importance of a multidisciplinary and biopsychosocial approach when dealing with cardiovascular patients and their problems (Griffo et al., 2008; Greco et al., 2014).

According to this new integrated vision, the role of the psychologist in the medical field is becoming increasingly necessary and advisable to grant to the patients a full and complete recovery, after critical cardiac pathologies.

The European Guidelines of 2016 support the importance of the evaluation and the treatment of psychosocial factors, in order to improve the QoL of patients and their prognosis (Piepoli et al., 2016).

In support of this new perspective, recent studies have shown how psychosocial factors, such as anxiety, depression, QoL, relationships and social status, adherence to drugs, smoking, drugs, alcohol, education, sleep, religiosity, work, nutrition, and physical activity can affect the recovery of patients after a cardiac surgery (Fattirolli et al., 2018).

In addition to the crucial cardiac rehabilitation phase for the full psycho-physical recovery of patients, the pre-operative phase is considered an equally delicate phase, during which patients are particularly vulnerable from a psychological point of view. The pre-operative period represents a possibility of cure, but at the same time also of failure. Fear of the unknown, together with this probability of failure, can aggravate the state of anxiety in which the patient finds themselves, causing further complications (Gomes et al., 2019).

An assessment and treatment of psychosocial risk factors both in the pre-operative phase in preventive terms and in the rehabilitation phase in terms of treatment can therefore improve the QoL of patients, and for this reason, it would be an ethical obligation to provide a complete and effective psychosocial treatment (Pogosova et al., 2015).

## Limitations

A limitation of our study concerns the fact that not all patients undergoing cardiac surgery are admitted for cardiac rehabilitation at IRCCS Policlinico San Donato; hence, the three time-period assessment will not always be possible for all patients. Data will be initially included from a single center, specifically investigating the hospitalization phase, but we hope that our protocol will be extended both on national and international levels.

## Conclusion

In Italy, the IRCCS Policlinico San Donato for its particular organizational structure, from 2013 allows psychologists to come into contact with patients before cardiac surgery. This organization makes it possible to identify the psychosocial characteristics of acquired cardiac patients from the pre-operative phase up to the discharge phase from rehabilitative cardiology, in the case of those patients who continue their rehabilitation process in the same institute. From what has been said so far, the close link between cardiovascular pathologies and psychological and psychosocial factors is evident. The use of a biopsychosocial and multidimensional approach is recommended to optimize and make cardiac care more effective. Based on the study results, the research protocol could be extended to a multi-centric level and to a European/Worldwide level.

## Data Availability Statement

The raw data supporting the conclusions of this article will be made available by the authors, without undue reservation.

## Author Contributions

EC proposed the study design and the idea, analyzed the literature and its development, and gave the final approval of the manuscript. SP and VF analyzed the literature, contributed to the study protocol, and prepared the manuscript. EGB contributed to the shaping of the manuscript and the psychodiagnostic tools selection. SB contributed to the statistical analyses. ACJ, CV, SC, MV, and LM have revised and approved the final manuscript. All authors contributed to the article and approved the submitted version.

### Conflict of Interest

The authors declare that the research was conducted in the absence of any commercial or financial relationships that could be construed as a potential conflict of interest.
